# Editorial: New insights into the cognitive neuroscience of attention

**DOI:** 10.3389/fnhum.2023.1338738

**Published:** 2023-11-29

**Authors:** Tetsuo Kida, Hidehiko Okamoto

**Affiliations:** ^1^Higher Brain Function Unit, Department of Functioning and Disability, Aichi Developmental Disability Center, Institute for Developmental Research, Kasugai, Japan; ^2^Department of Physiology, International University of Health and Welfare Graduate School of Medicine, Narita, Japan

**Keywords:** attention, neuroimaging, electroencephalography, magnetoencephalography, executive functions, working memory, oscillatory activity, near infrared spectroscopy (NIRS)

Attention is associated with the selective directedness of our mental lives. It has been extensively examined since the nineteenth century, and recent studies have elucidated the underlying neural mechanisms and functionality in living humans using various human neuroscientific techniques. This Research Topic is a collection of studies relevant to attention in health and disease using human neuroscientific techniques, including event-related brain potentials (ERPs) and magnetic fields (ERFs), oscillatory activity on magnetoencephalograms (MEG) or electroencephalograms (EEG), sheet-type wearable EEG devices, functional magnetic resonance imaging (fMRI), functional near-infrared spectroscopy (fNIRS), and novel methods for the measurement and modeling of attention.

EEG is a classical technique to measure human neurophysiological activity, and recent advances in technology and experimental paradigms have rendered EEG a unique and powerful tool (Michel and Murray, 2012; Kida et al., 2015). This Research Topic consists of a number of EEG-based studies; five are based on the ERP technique, which has long been used to elucidate the neural mechanisms underlying attention (Hillyard et al., 1998; Martinez et al., 1999; Eimer and Driver, 2001; Muller et al., 2003; Eimer et al., 2009; Jones and Forster, 2013; Forster et al., 2016; Gherri et al., 2016, 2023; Qin et al., 2022). Using ERPs, Rutkowska et al. investigated a working memory (WM)-based guidance effect for naturalistic, complex stimuli. WM-based guidance is the automatic capture of visual attention generated when a sensory stimulus matches a stimulus representation voluntarily stored in WM. Memorized items did not evoke a lateralized N2pc ERP component, which was considered to indicate attention shifts (Luck and Hillyard, 1994; Eimer, 1996; Eimer and Kiss, 2007). In contrast, ERP modulation for very early prioritization specific to memorized faces was noted, which was consistent with the sensory recruitment theory of WM. Therefore, complex stimuli were prioritized by attention when maintained in WM, and the mechanism underlying this prioritization was considered to be based on a prolonged hold of spatial attention. Gherri et al. identified a reliable N140cc component (Forster et al., 2016), a somatosensory homolog of N2pc, for all set sizes in a tactile search task, and the N140cc amplitude decreased as the number of distractors increased. The addition of distractors appeared to impede the preattentive processing of the search array, resulting in increased uncertainty about the target location. This, in turn, increased the variability of directing attention to the target, resulting in reduced N140cc amplitudes. These findings indicated distinct patterns of attention between touch and vision. A study by Kimura and Katayama used ERP as an index of attention and prediction error and event-related spectral perturbation as an index of prediction to elucidate the predictive function of the visuo–tactile interaction in the peri-personal space. Their findings showed that approaching visual stimuli facilitated the prediction of subsequent tactile events regardless of whether they were relevant to the task. An ERP study by Kida et al. demonstrated the effects of focused and divided attention on N140 and P300 in somatosensory selective attention tasks with different response modes (motor response and silent counting), and also attentional modulation under different hand and arm postures. They also revealed attentional alterations in stimulus-response coupling using a single-trial analysis of the P300 latency and reaction time, which showed different patterns from data for dual-task vs. single-task conditions (Kida et al., 2012). An auditory ERP study by Sasaki et al. showed that the amplitude of phonological mapping negativity and N400, which are objective measures of phonological and semantic processing, respectively, increased when the picture of an event and speech mismatched. The authors suggested that established sound symbolic words and sound symbolic pseudowords undergo similar semantic processing. The ERP technique has long been used to elucidate the mechanisms underlying attention, and the findings published here indicate that this technique is still effective for that purpose.

Other studies published here using EEG proved its diverse usefulness for examining and estimating attentional states. Wan et al. measured EEG during the performance of a sustained attention task and in a resting state. A new classification model, Complexity-XGBoost, was proposed using EEG-based dynamical complexity features and Extreme Gradient Boosting (XGBoost) to discriminate multi-level attention states with greater accuracy. The proposed model outperformed the other classification methods, leading the authors to conclude the value of the proposed approach for classifying the attention status in order to improve safety and efficiency, and also its applicability to the brain-computer interaction. Dini et al. measured EEG while participants watched videos with high and low levels of narrativity, and calculated the inter-subject correlation of EEG and attentional engagement scores. High-level narrativity was associated with higher inter-subject correlation and attention scores, suggesting that the findings obtained are a step toward elucidating the viewers' way of processing. Callan et al. measured EEG gamma and alpha oscillations in association with inattentional deafness using a wireless EEG device. In an auditory detection task, task performance (hits and misses) was associated with alpha- and gamma-band activities, respectively, in different hemispheres of the auditory cortex. Additional activities were detected in the frontal and parietal regions that reflected attentional monitoring, selection, and switching. Therefore, these findings showed the involvement of gamma and alpha activities in frontal and modality-specific regions in selective attention. Ueno et al. used a sheet-type wearable EEG device to measure the frontal midline theta rhythm (fmθ), which emerges during the attentional focus state, in a resting state and mental calculation task. Participants with fmθ showed higher gamma and theta power and lower alpha power, leading the authors to conclude that gamma activity in the frontal region was associated with WM.

This Research Topic was also contributed to by other neuroscientific techniques, such as MEG, fNIRS, genetics neuroimaging, and behavioral measures. EEG is a record of extracellular volume currents (secondary current) that originate from the current source and flow in the brain, whereas MEG is a record of the in/outflux of magnetic fields generated following intracellular currents (primary current; Hamalainen et al., 1993; Kida et al., 2015). The magnetic field is highly permeable in the skull, scalp, meninges, and cerebrospinal fluid and, thus, MEG has advantages for accuracy and the computational demand of source localization. MEG studies reported attentional modulations using event-related (evoked) activity in audition (Okamoto et al., 2007), vision (Kida et al., 2011), and touch (Kida et al., 2018) as well as oscillatory activity (Siegel et al., 2008). In this Research Topic, Onishi and Yokosawa used MEG to examine theta-band oscillatory activity during a WM task. Theta activity exhibited a dynamic pattern from attentional control and inhibition control in the prefrontal cortex to the inhibition of task-irrelevant information in the occipito-parietal cortex. An fNIRS study by Ohshima et al. examined the neural correlates of executive functions (EFs) based on an emotional vocal cue in an N-back task. The findings obtained showed that the right precentral and inferior frontal gyri were activated during the emotional N-back task, reflecting the function of an attentional network with auditory top-down processing. Significant activation was also detected in the ventrolateral prefrontal cortex, an important area for WM. Therefore, this study demonstrated the neural correlate of emotional judgments based on vocal cues in comparison with that for gender judgments. Huang et al. reported genetics neuroimaging in attention deficit/hyperactivity disorder (ADHD), one of the most common neurodevelopmental disorders. The study performed a functional connectivity (FC) analysis of resting-state fMRI data in 60 ADHD patients and 28 healthy controls, and genotyped single nucleotide polymorphisms in synaptosomal-associated protein-25 (SNAP-25) to separate patients into two groups: TT homozygotes and G-allele carriers. In comparisons with the TT group, the TG group showed significant changes in FC between the hippocampus and other regions. The authors proposed that hippocampal FC may serve as an imaging biomarker for ADHD. Younger et al. developed a novel behavioral measure and modeling of EFs closely associated with attentional functions, and used a large-scale diverse dataset in middle childhood to reveal the evolving diversity of EFs, including WM, context monitoring, and interference resolution, while simultaneously accounting for their unity. The findings obtained showed that the eight tasks were organized into three stable components by the age of 10 years, whereas refinements in the composition of these components continued through to at least the age of 14 years.

The novel analytical approaches, measurement devices, ERP components, and oscillatory activities discussed in this Research Topic will advance research on the neuroscience of attention. The variety of methodologies and themes represents the expansion of this research field, suggesting that the cognitive neuroscience of attention will continue to progress in the future.

## Author contributions

TK: Conceptualization, Writing – original draft. HO: Conceptualization, Writing – review & editing.
